# The Role of MicroRNAs in the Pathogenesis of Diabetic Nephropathy

**DOI:** 10.1155/2019/8719060

**Published:** 2019-12-01

**Authors:** Jian Tang, Deyi Yao, Haiying Yan, Xing Chen, Linjia Wang, Huakui Zhan

**Affiliations:** ^1^Hospital of Chengdu University of Traditional Chinese Medicine, Chengdu 610075, Sichuan, China; ^2^Chengdu University of Traditional Chinese Medicine, Chengdu 610075, Sichuan, China

## Abstract

Diabetic nephropathy (DN) is one of the most common microvascular complications in diabetic patients; it is also an important cause of renal dysfunction, renal fibrosis, and end-stage renal disease. Unfortunately, the pathogenesis of DN is complex and has not yet been fully elucidated; hence, the pathogenesis of DN to determine effective treatments of crucial importance is deeply explored. Early DN research focuses on hemodynamic changes and metabolic disorders, and recent studies have shown the regulatory role of microRNAs (miRNAs) in genes, which may be a new diagnostic marker and therapeutic target for diabetic nephropathy. In this review, we summarize the recent advances in the clinical value and molecular mechanisms of miRNAs in DN, providing new ideas for the diagnosis and treatment of DN.

## 1. Introduction

Diabetic nephropathy (DN) is a progressive kidney disease secondary to diabetes and has been shown to be a major cause of end-stage renal disease (ESRD) [1], accounting for nearly 30%–50% of the world's population requiring renal replacement therapy [2, 3]. As we all know, DN is the result of a combination of factors, for example, genetic susceptibility, glucose metabolism disorder, renal hemodynamic changes, oxidative stress, and cytokines all play a very important role [4]. Renal function and structural changes are the pathological features of DN, including albuminuria, glomerular and tubular hypertrophy, glomerular basement membrane thickening, renal interstitial fibrosis, and podocyte injury [5, 6]. Moreover, the degree of renal fibrosis which was considered to be a key indicator of worsening kidney function is also the core of DN high mortality [7], mainly due to the accumulation of extracellular matrix (ECM) proteins (e.g., collagen and fibronectin), as well as epithelial-to-mesenchymal transition (EMT) [8, 9]. At present, microalbuminuria is recognized as the gold standard for the diagnosis of DN. Early appearance of microalbuminuria in patients with DN, with the progress of the disease, will cause significant proteinuria, impaired renal function, glomerular filtration rate (GFR) gradually decreased, eventually leading to ESRD [10]. In recent years, a large body of research shows that miRNAs participate in regulating vital biological processes, for instance, multiplication, polarization, apoptosis, and metabolism [11], which are applicable to potential new biomarkers for a variety of diseases. Similarly, special miRNAs regulate the pathophysiology processes of DN by answering different signaling pathways and acting on different targets to inflammatory response, oxidative stress, immune response, fibrosis, and cell function.

## 2. MicroRNAs

MiRNAs are a class of noncoding single-stranded small RNA molecules of about 22 nucleotides in length [12]. MiRNAs regulate the expression of target genes by incompletely pairing with the base of the 3'-untranslated region (3'-UTR) of the target mRNA, and its specific regulation includes inhibition of mRNA translation and interference with mRNA stability [12, 13]. According to the latest research, a number of significantly altered miRNAs have been detected in human tissues and biological fluids and can be easily assessed by sensitive and specific methods [14]. There is increasing evidence that the imbalance of miRNAs is involved in the proliferation and invasion of tumor cells, autoimmune diseases, cardiovascular disorders, and the progression of DN [6, 15]. MiRNAs play an important role in multiple pathogenesis of DN, for example, glomerular basement membrane (GBM) and mesangial pathological changes and ECM accumulation, a hallmark of renal tissue fibrosis. For instance, in mesangial cells treated with high glucose, overexpression of microRNA-141 aggravates cell inflammation and promotes cell apoptosis [16]. MicroRNA-93 overexpression prevented transforming growth factor- (TGF-) *β*1-stimulated EMT and renal fibrogenesis by targeting Orai1 expression [9]. Therefore, it is possible for miRNAs to become diagnostic biomarkers and therapeutic targets for DN.

## 3. MicroRNAs Upregulated in DN

### 3.1. MiR-21

MiR-21 is a multipotent miRNA that has been frequently studied to promote cell proliferation, inflammation, angiogenesis, and immune destruction [17]. In recent years, studies have confirmed that miR-21 is one of the most important microRNAs involved in renal fibrosis, and its level is upregulated in renal tissues [18]. Studies in different periods have found that overexpression of miR-21 inhibits the proliferation of mesangial cells under high-glucose conditions, while increasing Akt activation induces mesangial cell hypertrophy and fibronectin expression [19, 20]. During the kidney fibrosis, the degree of glomerular fibrosis was positively correlated with the expression level of miR-21. Overexpression of miR-21 enhances TGF-*β*1-induced EMT by downregulating smad7 and upregulating smad3 expression. More importantly, miR-21 inhibitors not only prevent progression of EMT and renal fibrosis but also improve kidneys structure and function in diabetic nephropathy [21, 22]. In conclusion, suppression of miR-21 may be an effective target to directly reduce renal fibrosis in DN.

### 3.2. MiR-34a-5p

Tubulointerstitial fibrosis (TIF) is an important indicator of the severity of renal insufficiency, so it is of great significance to elucidate the pathogenesis of TIF in DN. In a recent study, it is reported that miR-34a-5p directly targets SIRT1 (SIRT1 is one of the silencing regulatory protein complexes that blocks DNA transcription at its site) to take part in TIF during DN [23]. In the kidney tissues of DN mice, the level of miR-34a-5p is upregulated and the expression of SIRT1 is decreased, low expression of SIRT1 results in a significant increase in TGF-*β*1 expression [24]. These results suggest that miR-34a-5p/SIRT1 may aggravate the progression of TIF in DN through TGF-*β*1 signaling pathway [24]. Except for TIF, upregulation of miR-34a-5p regulates stearic acid-induced beta-cell lipid toxicity by inhibiting BCL-2 and BCL-W (antiapoptotic members of the BCL-2 family) protein production [25]. Therefore, reducing miR-34a-5p may be an effective strategy for preventing and treating TIF and *β*-cell lipotoxicity.

### 3.3. MiR-141

MiR-141 is a member of the miR-200 family (also included are miR-200a, miR-200b, miR-200c, and miR-429), encoded by chromosome 12 [26]. Previous evidence suggests that miR-141 is involved in cancer cell growth and apoptosis. Researchers collected serum from DN patients and healthy subjects to analyze the relationship between miR-141 level and degree of renal injury and found out that patients with high levels of miR-141 have severe renal pathological damage. DN is also considered an inflammatory disease since significantly upregulated levels of inflammatory cytokines promoted the development of DN [27]. Li et al. [16] confirmed through experiments that the expression of miR-141 in peripheral blood of patients with DN was significantly increased; in the cell model of DN, overexpression of miR-141 aggravates cell inflammation and promotes cell apoptosis. In addition, there may be a targeting relationship between insulin receptor substrate 2 and miR-141. By contrast, many investigators found that miR-141 is underexpressed in the kidney tissue of the DN model, and downregulation of miR-141 specifically induces TGF-*β*2 to promote renal scar formation [28, 29]. These results indicate that miR-141 can be used as a novel biomarker for controlling blood glucose and preventing DN renal fibrosis.

### 3.4. MiR-370

MiR-370 is located in the DLK1/DIO3 region of chromosome 14, with credible evidence suggesting that it can regulate tumorigenesis and lipid metabolism [30, 31]. When Yu et al. [32] explored the association between miR-370 and diabetic nephropathy, their results showed that the expressions of miR-370, fibronectin, type I collagen (Col I), type IV collagen (Col IV), and plasminogen activator inhibitor-1 (PAI-1) increased in DN rats, and changes in the expression of these molecules affect the polymerization and degradation of ECM [33, 34]. In addition, the proliferation of mesangial cells decreased and the accumulation of ECM decreased after the addition of the miR-370 inhibitor. Thus, inhibition of the expression of mir-370 may be a new method for the treatment of DN.

### 3.5. MiR-503

Podocyte and vascular endothelial cell injury play a key role in the pathogenesis of DN. Research data showed that miR-503 was involved in diabetic endothelial dysfunction, and the expression of miR-503 in limb muscles of diabetic patients increased sharply. Meanwhile, antagonizing miR-503 using adenovirus-mediated locally delivered competitive inhibitors improved postischemic repair of neovascularization and blood flow recovery [35]. Overexpression of miR-503 also causes podocyte injury, which results in adverse kidney damage in DM rats by targeting E2F3, while inhibition of miR-503 reduced podocyte injury [36]. Losartan is used successfully in the treatment by inhibiting the expression of mir-503 in KKAy mice and improved the development of DN. More surprisingly, in treatment of diabetic cardiomyopathy with Phase II enzyme inducer (CPDT), miR-503 was decreased, myocardial cell apoptosis was decreased, and the myocardial systolic and diastolic functions were improved [37, 38]. Overall, downregulation of miR-503 protects diabetic podocyte and vascular endothelial cells.

### 3.6. MiR-184

Recent studies have found a close relationship between miR-184 and renal fibrosis. When studying the miRNA profile of DN, Zanchi et al. [39] found that miR-184 showed the most significant differential upregulation in the kidneys of DN late Zucker diabetic fatty (ZDF) rats. In addition, Zanchi's team also found that the expression of miR-184 was concentrated in the damaged proximal tubule region and was associated with decreased expression of lipophosphatase 3 (LPP3) and accumulation of collagen. A decrease in LPP3 increases the expression level of lysophosphatidic acid (LPA), and studies have reported that an increase in LPA release is associated with tubulointerstitial fibrosis [40, 41]. Coincidentally, Zanchi et al. also found a reduction in LPP3 staining in serial kidney sections. It is well known that albuminuria is an important stimulator of renal fibrosis affecting diabetic nephropathy. In experiments, Cristina et al. exposed rat NRK-52E cells to angiotensin II, albuminuria, and TGF-*β* and found that albuminuria is the main effective inducer of miR-184, while angiotensin II expression of miR-184 in NRK-52E cells could not be induced [39]. More importantly, the NF-*κ*B binding site is present in the promoter of miR-184, which regulates inflammatory responses and fibrogenesis by activating NF-*κ*B signaling. Similarly, the use of ACE inhibitors in ZDF rats reduced miR-184 and retained LPP3 in the renal tubules, reduced proteinuria, and improved tubulointerstitial fibrosis. Furthermore, Liu et al. reported that miR-184 was significantly increased in rat senile glomerular mesangial cells, suggesting that kidney senescence is also associated with miR-184 [42]. In conclusion, these studies suggest that miR-184 is involved in renal tubulointerstitial fibrosis in DN and provides a theoretical basis for studying the targeting of miR-184 in combination with albuminuria to reduce DN fibrosis.

### 3.7. MiR-377

We mentioned in the foregoing that fibronectin is an excessive accumulation of ECM proteins in DN. Wang et al. [43] found that miR-377 is upregulated in DN, and overexpression of miR-377 can target the inhibition of the synthesis of some important mesangial cell proteins such as, PAK1, superoxide dismutase1 (SOD1), and superoxide dismutase2 (SOD2). However, a decrease in PAK1, SOD1, and SOD2 proteins leads to an increase in fibronectin accumulation, and in addition, an increase in miR-377 expression enhances oxidative stress in mesangial cells. Therefore, the inhibition of miR-377 expression is a new approach to the treatment of DN. Duan et al. [44] showed that long noncoding RNA taurine-upregulated gene 1 (lncRNA TUG1) can act as an endogenous miRNA sponge of miR-377, downregulating the expression level of miR-377, thereby attenuating the inhibition of its target gene peroxisome proliferator-activated receptor *γ* (PPAR*γ*); PPAR*γ* is associated with mesangial cell proliferation, cell cycle, and glomerular ECM synthesis in diabetic environment [45]. In general, miR-377 plays a key role in the development of DN, and the use of LncRNA to regulate miRNA expression is a novel treatment for DN.

## 4. MicroRNAs Downregulated in DN

### 4.1. Let-7 Family

Let-7 was first discovered in Caenorhabditis elegans, and let-7 is the most abundant of the miRNAs, with 11 members in humans [46, 47]. Supposedly, the miRNAs of the let-7 family have similar functions because they share a common seed region (nucleotides 2–8). Let-7 has been widely studied as a tumor suppressor; subsequent studies have supported the let-7 family as a potential target for regulating blood glucose and insulin in patients with type 2 diabetes [48]. Furthermore, the expression of the let-7 family is inhibited in DN and may increase again after improved glycemic control [49]. Recently, abnormal DNA methylation levels of miRNAs in the promoter region are also closely related to DN, for example, the expression of let-7a-3 is decreased in DN patients, while the DNA methylation level of let-7a-3 promoter is increased. Low expression of let-7a-3 and promoter hypermethylation can participate in the development of DN by targeting UHRF1/DNMT1 [50]. Also, there are many reports related to DN in the let-7 family, for example, the upregulation of let-7c can inhibit the renal fibrosis induced by TGF-*β*1, and the decrease of let-7a-5p promotes apoptosis of mesangial cells under high-glucose conditions [6, 48].

### 4.2. MiR-25

It is well known that enhanced oxidative stress is a common cause of diabetic microvascular complications. Available data suggest that diabetic patients have low levels of miR-25 in peripheral blood compared to nondiabetic patients; low levels of miR-25 decrease the silencing effect on the NADPH oxidase 4 (Nox4) gene in mesangial cells and result in increased production of reactive oxygen species (ROS) [51, 52]. Upregulation of miR-25 in diabetes can reverse DN kidney changes and reduce hypertension, as well as, miR-25 can induce renal failure through the Ras signaling pathway [53]. Accumulating evidences indicate that miR-25 functions as an anti apoptotic factor in many types of cells. For HG-induced renal tubular epithelial cells, miR-25 can improve oxidative stress and apoptosis by activating the PTEN/Akt pathway [54]. Hence, miR-25 can be used as an antioxidant and adjuvant therapeutic target for diabetic complications.

### 4.3. MiR-29 Family

The MiR-29 family contains three members, miR-29a, miR-29b, and miR-29c, and their binding targets are the same target gene including ECM gene loci Col1a1, 3a1, and other 9 sites [55]. TGF-*β*1 induced ECM protein over deposition, and EMT advances in the progression of DN fibrosis; studies have found that TGF-*β*1 can regulate the expression of some miRNAs. High expression of TGF-*β*1 can activate Smd3 and promote its binding with miR-29, resulting in low expression of miR-29; therefore, the miR-29 family is an important downstream mediator for TGF-*β*1-mediated fibrogenesis [55, 56]. Wang et al. demonstrated that miR-29a, miR-29b, and miR29c were reduced in all kidney disease models in early and late stages of renal fibrosis. And, the decreased expression of miR-29 was associated with increased expression of collagen and other ECM proteins. Moreover, the expression of the miR-29 family was also significantly reduced in the nondiabetic renal fibrosis models [56, 57].

### 4.4. MiR-93

MiR-93 is encoded by intron 13 of the MCM7 gene; it has been reported that miR-93 is a metabolically regulated miRNA and is differentially downregulated in the kidney of the diabetes model. Besides, metabolically regulated miR-93 plays a metabolic/epigenetic role in the remodeling of nucleosomes in the diabetic environment [58]. MiR-93 also indirectly maintains glomerular angiogenesis and vascular homeostasis by regulating the expression of VEGF *in vivo*. Overexpression of miR-93 eliminated VEGF downstream targets such as collagen IV and fibronectin [59]. As expected, miR-93 was significantly reduced in renal tissue of DN patients and in TGF-*β*1-stimulated HK2 cells, but miR-93 overexpression prevented TGF-*β*1-stimulated EMT and renal fibrogenesis by targeting Orai1 expression [9]. It indicates that miR-93 has antiangiogenic and antifibrotic effects.

### 4.5. MiR-126

MiR-126 is one of the most widely studied miRNAs in diabetes, and many studies have shown that the level of miR-126 in the blood circulation of patients with type 2 diabetes and DN is reduced [60, 61]. Recent studies have found that miR-126 is correlated with renal injury and endothelial aging and furthermore negatively correlated with the degree of blood glucose control [62, 63]. MiR-126 is abundantly enriched in endothelial cells and can enhance vascular endothelial growth factor (VEGF) signaling, which plays an important role in vascular protection and angiogenesis [64]. The results of several research experiments indicate that miR-126 is reduced in high-glucose-induced human glomerular mesangial cells (HGMCs). Upregulation of miR-126 mimics transfected with HGMCs delayed the premature aging of HGMCs may be related to prolonging the telomere length of HGMCs or inhibiting the activity of JAK/STAT signaling pathway *in vitro* [64, 65]. Besides, miR-126 additionally controls vascular inflammation through targeting and suppressing vascular cell adhesion molecule-1 (VCAM-1) and reduces the adhesion of leukocytes to endothelial cells [14, 66].

### 4.6. MiR-130b

MiR-130b is located in the intron of a noncoding RNA-2610318N02RIK (RIK). Recent studies have found that TGF-*β* can regulate the expression of the RIK gene, thereby downregulating miR-130b and increasing the expression of pathological profibrotic genes [67]. Several researchers found that plasma miR-130b decreased in DN patients, which was associated with decreased renal function. MiR-130b has been shown to play a role in oxidative stress, insulin resistance, lipid metabolism, and EMT. For instance, miR-130b can regulate the expression of Snail, the most mature major regulator of EMT [68–70]. Previous studies have shown that the expression level of serum miR-130b gradually decreases with the increase of urinary protein level in diabetic patients; thus, the miR-130b level can be considered as a good predictor of DN [71].

### 4.7. MiR-424

In the past, miR-424 was considered to have antioxidant and anti-inflammatory effects in diabetic patients and miR-424 was decreased in diabetic patients [72, 73]. Wang et al. [74] observed that the level of miR-424 in kidney tissue of type 1 diabetic nephropathy rats was significantly lower than that of the normal group. After treatment with miR-424 mimics, the symptoms of renal pathological changes and blood glucose and urine protein levels in DN rats were significantly improved. In a recent study, it was found that the upregulation of miR-424 may inhibit the apoptosis of renal tissue cells and reduce the pathological changes, and this process depended on the caspase-3 pathway, resulting in the adjustment of Bax and Bcl-2 levels [73].

### 4.8. MiR-146a

Studies have reported that miR-146a is highly expressed in podocytes and plays an important role in maintaining podocyte health [75]. Lee et al. [76] found that miR-146a expression was significantly reduced in glomeruli of diabetic patients and diabetic animals, and that decreased expression of miR-146a was associated with glomerular damage. Patients with lower glomerular miR-146a levels showed a significantly faster decline in renal function compared to patients with different miR-146a levels. Lee et al. also found that the expression of Notch-1 and ErbB4 in the glomerular direct targets of miR-146a was upregulated (both Notch-1 and ErbB4 are important developmental proteins). Interestingly, miR-146a disrupts ErbB4 signaling by inhibiting ErbB4 levels in podocytes; however, the diabetic environment activates certain pathways by TGF-*β*1 to decrease miR-146a levels and activate ErbB4 signaling. When diabetic patients use the known ErbB4 blocker erlotinib, it is found that proteinuria develops slowly and significantly reduces glomerular damage [76]. Moreover, the results of Wan et al. showed that increased expression of miR-146a inhibited inflammatory responses and oxidative stress in DN [77]. In conclusion, the protection of podocytes by miR-146a has a novel effect on the treatment of DN.

## 5. Urinary Exosomal MicroRNAs in DN

Urine is an ideal source of biomarkers for the diagnosis of renal disease; recent studies have found that urine contains exosomes, a discoid vesicle of 40–100 nm in diameter, which are usually secreted by all nephron cells and carry proteins, miRNAs, and other markers related to kidney damage [78–80]. More importantly, urinary exosomal microRNAs are very stable and are not easily confused with plasma miRNAs that pass through the glomerular filtration barrier [81].

Analysis of urinary exosomal miRNAs in patients with type 2 diabetes found that elevated levels of microRNA-192 and microRNA-215 aggravated kidney damage [82]. Xie et al. [83] found that 203 upregulated miRNAs and 188 downregulated miRNAs were associated with type 2 diabetes when extracting urine exosomes, with increased expression of microRNA-877-3p (miR-877-3p) and reduced expression of microRNA-15a-5p (miR-15a-5p). Studies have also found that miR-877-3p and miR-15a-5p mediate fibrosis in DN, and they are differentially expressed in DN and DM patients, which may be related to the fact that miR-877-3p is involved in oxidative stress to cause renal tubular cell apoptosis or increase the phosphorylation and transcriptional inactivation of FoxO3a through the PI3K/Akt pathway, so as to accelerate the development of renal disease [84].

It is known that microRNA-1915-5p (miR-1915-5p) is upregulated in the urine expression of DN patients, and miR-1915-5p can regulate TLR2 to promote renal tubular cell damage and inhibit renal repair mechanisms [85, 86]. TGF-*β* is the key mediator of renal fibrosis; microRNA-192 (miR-192), miR-29, and miR-200 families are regulated by TGF-*β*. MiR-192 increased expression in DN patients with renal fibrosis, while miRNAs in urine exosomes in diabetic patients and patients with early DN were not upregulated [87]. Instead, miR-29 levels in urinary exosomes were significantly downregulated, while treatment with the DPP-4 inhibitor (linagliptin) leads to restored miR-29 levels [88, 89]. In short, some of the imbalances in the levels of specific urinary exocytosis miRNAs can reflect renal fibrosis and may also be a way to diagnose DN staging.

## 6. MicroRNAs as Therapeutic Targets in DN

MiRNAs can directly target signaling pathways and specific pathways affecting the pathogenesis of DN. This suggests that the use of specific miRNA antagonists to silence the corresponding miRNAs *in vivo* may be an effective method for the treatment of DN. Many investigators have found that miR-192 is upregulated in DN and promotes collagen accumulation in mesangial cells by targeting E-box repressors (such as Zeb1 and Zeb2) [90]. Putta et al. observed that treatment of diabetic mice with locked nucleic acid-modified inhibitor of miR-192 (named LNA-anti-miR-192) was very effective in inhibiting the level of miR-192 [91]. Moreover, the fibrosis genes (Col1a2, Col4a1, and TGF-*β*) and connective tissue growth factors are simultaneously reduced. LNA-anti-miR-192 also attenuates glomerular expansion and renal fibrosis and reduces proteinuria in diabetic mice. Therefore, LNA-anti-miR-192 can be used to inhibit miR-192 treatment of DN.

Overall expression profiling revealed that miR-21 is one of the major miRNAs upregulated in the kidney of DN patients. Studies have shown that pharmacological silencing of miR-21 can significantly improve renal disease progression by stimulating metabolic pathways in a mouse model of Alport disease [92]. Kölling et al. found that miR-21 is enriched in glomeruli in DN and that cell division cycle 25a (Cdc25a) and cyclin-dependent kinase 6 (Cdk6) are novel targets for miR-21 [93]. MiR-21 inhibits cell division by targeting Cdc25a and Cdk6, which in turn leads to mesangial cell hypertrophy. Subsequently, Kölling et al. used a locked nucleic acid targeting miR-21 to treat DN, and proteinuria, interstitial fibrosis, and inflammatory symptoms were well improved. Moreover, no serious adverse events were shown in phase I clinical trials using miR-21 antagonists in healthy volunteers. In general, pharmacologically effective silencing of miRNAs would be a novel therapeutic strategy for the future treatment of DN.

## 7. Conclusion

Currently, miRNAs have been widely used as biomarkers in clinical diagnosis and treatment of DN. Specific changes of miRNAs in renal tissue, peripheral blood, and urine affect the occurrence and development of DN by regulating inflammatory response, oxidative stress, metabolic abnormalities, immune response, and fibrosis through different signal pathways and targets. This article mentions that some microRNAs show high and low expression in DN, of course, the imbalance of microRNAs expression level is not the only factor in the development of DN, but it plays a certain role in the regulation of DN development and prognosis. At the same time, correcting the imbalance of microRNA expression levels has a clear therapeutic effect on DN. However, there are still many challenges related to the research and clinical application of miRNAs and DN. Most studies have not established appropriate disease controls to determine whether these miRNAs are specific to DN or general markers of renal damage, so it is necessary to search for some highly specific miRNAs. Meanwhile, there is still a lack of prospective validation, large sample size, and multicenter and multiethnic patient studies on miRNAs related to DN diagnosis. In short, the emergence of miRNAs provides new ideas for the diagnosis and treatment of DN, and we need to conduct more studies to further understand the mechanism of microRNAs in DN.
